# Rapid inflammasome activation in microglia contributes to brain disease in HIV/AIDS

**DOI:** 10.1186/1742-4690-11-35

**Published:** 2014-05-13

**Authors:** John G Walsh, Stacey N Reinke, Manmeet K Mamik, Brienne A McKenzie, Ferdinand Maingat, William G Branton, David I Broadhurst, Christopher Power

**Affiliations:** 1 Department of Medicine (Neurology), Heritage Medical Research Centre 6–11, University of Alberta, Edmonton T6G 2S2, Canada

**Keywords:** Inflammmasome, NLRP3, IL-1beta, HIV-1, FIV, Caspase-1, ASC microglia, Nervous system

## Abstract

**Background:**

Human immunodeficiency virus type 1(HIV-1) infects and activates innate immune cells in the brain resulting in inflammation and neuronal death with accompanying neurological deficits. Induction of inflammasomes causes cleavage and release of IL-1β and IL-18, representing pathogenic processes that underlie inflammatory diseases although their contribution HIV-associated brain disease is unknown.

**Results:**

Investigation of inflammasome-associated genes revealed that IL-1β, IL-18 and caspase-1 were induced in brains of HIV-infected persons and detected in brain microglial cells. HIV-1 infection induced pro-IL-1β in human microglia at 4 hr post-infection with peak IL-1β release at 24 hr, which was accompanied by intracellular ASC translocation and caspase-1 activation. HIV-dependent release of IL-1β from a human macrophage cell line, THP-1, was inhibited by NLRP3 deficiency and high extracellular [K^+^]. Exposure of microglia to HIV-1 gp120 caused IL-1β production and similarly, HIV-1 envelope pseudotyped viral particles induced IL-1β release, unlike VSV-G pseudotyped particles. Infection of cultured feline macrophages by the related lentivirus, feline immunodeficiency virus (FIV), also resulted in the prompt induction of IL-1β. *In vivo* FIV infection activated multiple inflammasome-associated genes in microglia, which was accompanied by neuronal loss in cerebral cortex and neurological deficits. Multivariate analyses of data from FIV-infected and uninfected animals disclosed that IL-1β, NLRP3 and caspase-1 expression in cerebral cortex represented key molecular determinants of neurological deficits.

**Conclusions:**

NLRP3 inflammasome activation was an early and integral aspect of lentivirus infection of microglia, which was associated with lentivirus-induced brain disease. Inflammasome activation in the brain might represent a potential target for therapeutic interventions in HIV/AIDS.

## Background

Despite the success of antiretroviral therapies (ART), human immunodeficiency virus type 1 (HIV-1) infection remains a major global health challenge for which the pathogenic mechanisms resulting in the acquired immunodeficiency syndrome (AIDS) and its associated complications remain incompletely understood. HIV-1 enters the central nervous system (CNS) during primary infection and productively infects brain macrophage cell types including microglia and infiltrating macrophages [1]. Indeed, all of the immunosuppressive lentiviruses, including human (HIV), simian (SIV), bovine (BIV) and feline (FIV) immunodeficiency viruses, share the property of efficiently infecting both macrophage and lymphoid cells. This shared cell tropism contributes to chronic immune disease and eventual CNS disease during infection by each of these viruses, as evidenced by neuronal injury and neurological disabilities (motor/gait impairments, memory deficiencies, executive/behavioral abnormalities) [2]. However, the primary pathogenic event(s) underlying neuronal injury and death during lentivirus infections remain uncertain. CNS-associated disease represents a substantial burden among HIV-1 infected individuals because of the brain’s privileged immune status together with limited accessibility of antiretroviral therapies [3]. The prevalence of HIV-associated neurocognitive disorders is reported to be approximately 20-50% among treated populations [4,5]. The development of HIV-induced brain disease is characterized by inflammation involving the induction of cytokines, chemokines, proteases and free radicals with ensuing neuronal injury and death [1,3,6].

Inflammation within the brain is a highly orchestrated response by the immune system to infections or non-infectious disorders. There is a burgeoning growth in information regarding the composition and functions of the brain’s innate immune system, largely implicating microglia, trafficking macrophages, and astrocytes [7-9]. Cells of the innate immune system express pattern recognition receptors (PRRs), which recognize molecular patterns on infectious agents or disease-associated host molecules. As part of an inflammatory response, some cytosolic PRRs are capable of forming complexes termed 'inflammasomes’, which direct the activation of caspase-1 through auto-proteolysis leading to the cleavage and subsequent release of IL-1β and IL-18 [10]. Inflammasome activation has been implicated in multiple infectious and immune diseases including those involving the brain [11-14]. The brain is sensitive to IL-1β and IL-18 signalling at both a systemic and local level because multiple cell types in the CNS express the receptors for these cytokines [15,16].

The innate immune response to HIV-1 is attenuated in some macrophage cell types through the actions of host molecules such as TREX1 or SAMDH1, which prevent sufficient accumulation of viral PAMPs within the cytosol to trigger a response [17,18]. Thus, many innate immune cell types exhibit restricted responses to HIV-1 infection. An exception is the plasmacytoid dendritic cell (pDC), which senses viral RNA through TLR7 and releases type 1 interferons [19-21]. Importantly, this is part of an immediate response to virus and does not require the establishment of productive infection [19,20]. Although the stimulatory capacity of free virus is much less than that of HIV-1 infected cells, both responses require uptake of virus into endosomes and in the context of free virus, uptake occurs independent of membrane fusion mediated viral entry [19-21].

IL-1β expression is elevated in the CNS during HIV-1, SIV and FIV infections [22-24]. Additionally, IL-1β has been reported to be released from monocyte-derived macrophages or from mixed glial cultures (human or rat) in response to live virus or soluble HIV-1 proteins [25-28]. Recent studies have also linked polymorphisms in the inflammasome gene *NLRP3* to an increased susceptibility to HIV-1 infection [29,30] and HIV-1 has been implicated in priming the NLRP3 inflammasome in macrophages [31]. Although these observations imply the formation of an inflammasome complex in response to HIV-1, this complex has not been explicitly examined in previous studies, particularly in the context of end-organ disease. These findings prompted us to hypothesize that expression and functions of inflammasome components contributed to the inflammatory response of CNS cells to HIV-1 and to the development of lentivirus-induced neurological disease.

Herein, we report on the expression of individual inflammasome components in the brains of patients with HIV/AIDS, chiefly in microglia, which was verified in cultured primary human microglia. In addition, exposure of primary human microglia or PMA-differentiated THP-1 cells to HIV-1 led to a rapid and short-lived release of IL-1β that was dependent on caspase-1 activation, K^+^ efflux and the NLRP3 inflammasome. Furthermore, the expression and predominance of inflammasome components and the participation of IL-1β in neuropathogenesis was confirmed using an *in vivo* model of lentivirus (FIV)-induced immunodeficiency and neurological disease.

## Results

### Inflammasome substrates and components are expressed in the brain during HIV-1 infection

**Figure 1:**
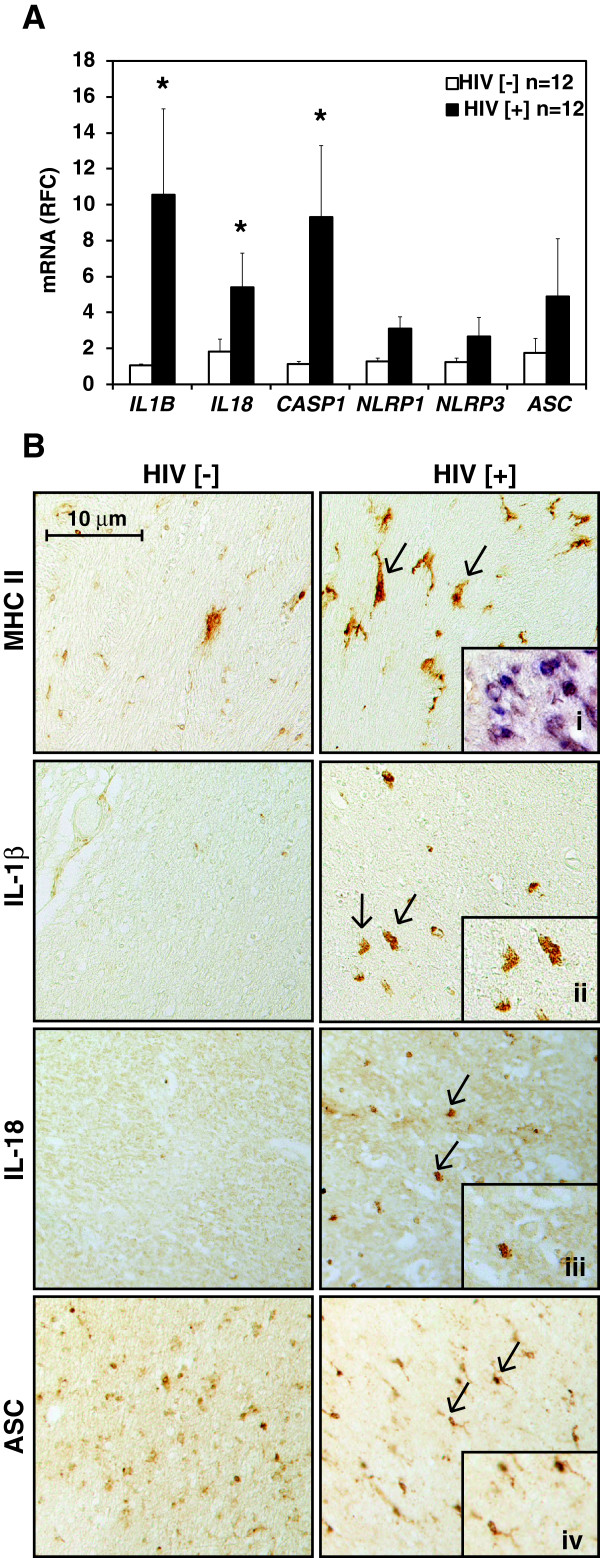
**Expression of inflammasome components and substrates in the HIV-1 infected human brain. A.** Relative fold change (RFC) in mRNA expression in the white matter of persons with HIV-1 infection (HIV [+]) (n = 12) compared to other disease controls (HIV [-]) (n = 12), as measured by semi-quantitative real-time PCR. Mean values are reported. Bars indicate standard error. *indicate p-value of <0.05. **B.** Immunohistochemistry staining of cerebral white matter sections from other disease control (HIV [-]) and HIV-infected (HIV [+]) (200X view). Inset 'i’ represents double immunostaining for MHC II and IL-1β. Insets 'ii’, 'iii’ and 'iv’ are magnified images of the indicated cells within the HIV [+] section.

Previous reports have highlighted increased IL-1β expression in the brains of HIV-infected persons [22]. To extend these studies, the expression of *IL-1B* and *IL-18* in conjunction with the inflammasome-forming nucleotide-binding oligomerization domain-like receptors (NLRs), *NLRP1* and *NLRP3*, as well as inflammasome components caspase-1 and apoptosis-associated speck-like protein containing a caspase recruitment domain (*ASC*) was examined in the cerebral white matter from HIV-infected (HIV [+]) and uninfected (HIV [-]) persons (Figure 1A). There was increased expression of caspase-1 (*CASP1*) and the inflammasome substrates, *IL-1*β and *IL-18,* in the brains of the HIV [+] group (p < 0.05) while the transcript expression of *NLRP1*, *NLRP3* or *ASC* was similar across groups (Figure 1A). To verify protein expression of inflammasome components, immunohistochemistry was performed on cerebral white matter sections from HIV [-] and HIV [+] persons, revealing minimal MHC Class II immunolabelling in sections from HIV [-] persons, while there was a marked increase in cells staining immunopositive for MHC Class II in the HIV [+] sections (Figure 1B). IL-1β immunoreactivity was not evident in HIV [-] brain sections but was detected in white matter from HIV [+] persons, with co-localization in MHC Class II immunopositive cells (Figure 1B, inset i) and in cells with activated microglial/macrophage morphology (Figure 1B, inset ii). Similarly, IL-18 immunoreactivity was negligible in HIV [-] white matter but detected within the white matter of HIV [+] persons (Figure 1B, inset iii). ASC immunoreactivity was observed in both groups, likely reflecting the constitutive nature of this protein (Figure 1B, inset iv). Thus, several components of the inflammasome exhibited increased expression in the brain during HIV-1 infection, largely in cells of macrophage lineage.

### Expression and activation of inflammasome genes in human primary CNS cell types

**Figure 2:**
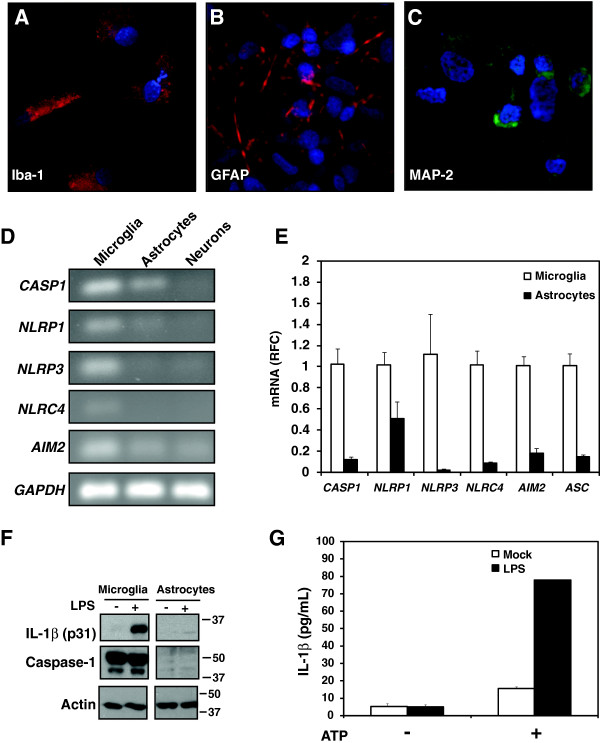
**Expression and activation of the inflammasome in primary human CNS cells.** Isolated primary human cells including microglia **(A)**, astrocytes **(B)** and neurons **(C)** were immunolabelled with antibodies to Iba-1, GFAP and MAP-2, respectively, to verify the purity of cell cultures. **D.** Qualitative RT-PCR showing transcript expression of caspase-1 as well as various inflammasome-forming cytoplasmic pattern recognition receptors in primary cultured human microglia, astrocytes and neurons. **E.** Semi-quantitative real-time PCR showing the relative fold change in inflammasome-associated gene expression in cultured primary human microglia and astrocytes. Bars indicate standard error. **F.** Protein expression of IL-1β and caspase-1 in primary human microglia but not astrocytes. **G.** IL-1β release from Mock-or LPS-primed (10 ng/mL, 6 hr) primary human microglia followed by ATP stimulation (5 mM, 1 hr). Mean values are reported. Bars indicate standard error.

Although different components of inflammasome machinery have been reported in the human brain, their specific cellular expression was uncertain. Using isolated primary human neural cells including Iba-1 immunopositive microglia (Figure 2A), GFAP immunopositive astrocytes (Figure 2B) and MAP-2 immunopositive neurons (Figure 2C), the mRNA expression of *CASP1, NLRP1, NLRP3, NLRC4* and *AIM2* was investigated in each cell type (Figure 2D). The purity of these primary cell cultures has been previously characterized by both our group as well as others [32-38]. Isolated microglia exhibited the highest expression of all the genes examined, while expression in astrocytes or neurons was either much lower or not detected (Figure 2D). In addition, a similar comparison of gene expression between primary microglia and astrocytes, by semi-quantitative real-time PCR confirmed these observations (Figure 2E). Of note, the expression of inflammasome components in primary human microglia and astrocytes was also compared with that of the human monocyte cell line, THP-1, revealing gene expression levels for THP-1 cells and primary microglia that were broadly similar while the expression of inflammasome-related genes in astrocytes was markedly less (Additional file 1: Figure S1).

Although gene expression studies suggested that, among the human CNS cells examined, microglia are best equipped with inflammasome components, astrocytes have been reported to express and/or release IL-1β upon activation by various stimuli including bacterial lipopolysaccharide (LPS) [39-41]. To compare the ability of primary human microglia and astrocytes to express IL-1β protein, human microglia and astrocytes were exposed to LPS. This stimulus induced robust IL-1β expression in microglia as evidenced by immunodetection of the pro-form of the cytokine (~31 kD) (Figure 2F). In addition, full-length caspase-1 (~45 kD) was constitutively expressed in microglia. However, neither IL-1β nor caspase-1 were detected in primary astrocytes even under conditions of *LPS* exposure (Figure 2F), implying that astrocytes are unlikely to be a major contributor to inflammasome-dependent IL-1β release within the brain.

Priming gene expression through NFκB activation is the first purported step (“Signal 1”) required for expression of the IL-1β pro-form. A second signal (“Signal 2”) is also required to trigger the formation of the inflammasome complex, which mediates the maturation and release of IL-1β from cells [42]. To confirm that human microglia would respond in a similar manner, cultures were primed with LPS (Signal 1) followed by stimulation with extracellular ATP (Signal 2), which is an established activator of the NLRP3 inflammasome. While LPS exposure alone did not induce release of IL-1β, subsequent ATP (5 mM) treatment triggered release of the cytokine, pointing to a conventional response of human microglia to NLRP3 activating paradigms (Figure 2G). These studies established that microglia were the chief sources of IL-1β in the human brain and were also the principal cells encoding components of the inflammasome.

### IL-1β expression and release from human microglia following HIV-1 infection

**Figure 3:**
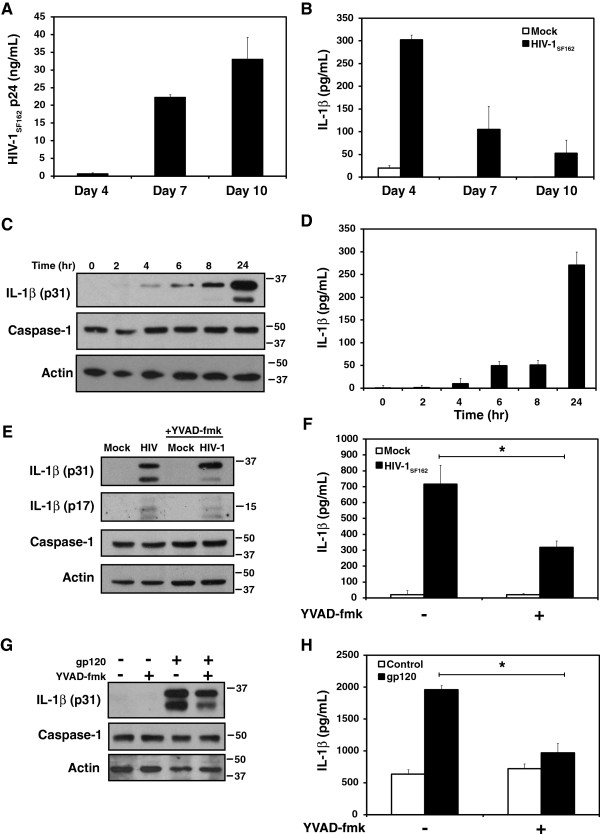
**IL-1β expression and release from primary human microglia following HIV-1 infection or exposure. A.** Viral p24 and **B.** IL-1β release from microglia at days 4, 7 and 10 following infection with HIV-1_SF162_.**C.** IL-1β induction and **D.** release from microglia cultures over 24 hr following exposure to HIV-1_SF162_. **E.** Induction, processing and **F.** release of IL-1β following 24 hr exposure to HIV-1_SF162_ +/- the caspase inhibitor YVAD-fmk (80 μM). **G.** Induction and **H.** release of IL-1β from microglia cultures exposed to recombinant gp120 (80 nM) for 24 hr +/- YVAD-fmk. Mean levels are reported for each ELISA. Bars indicate standard error. We compared the effect of HIV-1gp120 exposure on IL-1β release +/- YVAD-fmk using two-tailed Student *t*-test (*p < 0.05).

As several inflammasome components were induced in the brain during HIV-1 infection, the ability of HIV-1 to activate inflammasome-dependent IL-1β release was explored using primary human microglial cultures. Following infection of cultured microglia with the CCR5-dependent HIV-1 strain, HIV-1_SF162_, there was an observed release of HIV-1 p24 detected at day 4 post-infection, which increased at days 7 and 10 (Figure 3A). In contrast, IL-1β release from the same HIV-infected cells was highest at day 4 post-infection but declined at days 7 and 10 (Figure 3B). In addition, pre-treatment of cells with reverse-transcriptase inhibitors zidovudine (AZT) or efavirenz failed to block HIV-mediated expression of IL-1β in microglia (Additional file 1: Figure S1B). This observation suggested that very early HIV-1 exposure or infection promoted IL-1β release from cells; thus, the immediate response of microglia to HIV-1_SF162_ exposure was examined. Analyses of IL-1β expression in microglia following exposure to HIV-1_SF162_ showed induction of full length IL-1β (p31) by 4 hr post-infection increasing by 24 hr post-infection at which time the processing of IL-1β could be observed in the appearance of a 29 kD band, representing the first of two cleavage events that are required for the maturation of the cytokine [43,44] (Figure 3C). In addition, IL-1β release from HIV-1_SF162_-exposed microglia was evident at time points as early as 6 hours (Figure 3D). Caspase-1 expression was stable during HIV-1_SF162_ exposure (Figure 3C) although as is often reported it was not possible to observe processed caspase-1 in the cell lysates [45]. However, blocking caspase activity by applying the caspase inhibitor YVAD-fmk, prior to exposure with HIV-1_SF162_, inhibited both the processing (Figure 3E) and release (p < 0.05) (Figure 3F) of IL-1β from human microglia. In addition, an inhibitor of actin polymerization, cytochalasin D, also inhibited IL-1β release from human microglia, suggesting that virus might be sensed through an endocytic pathway (Additional file 1: Figure S1C). As soluble HIV-1 gp120 has been previously reported to induce IL-1β release from macrophage cells or glial cultures [25,27,28], this response was investigated in primary human microglia in the presence of YVAD-fmk; exposure of human microglia to soluble gp120 encoded by the CCR5-dependent HIV-1 CM235 strain, induced IL-1β expression (Figure 3G) and release (Figure 3H), which was significantly inhibited by YVAD-fmk.

### IL-1β release in response to HIV-1 is dependent on the NLRP3 inflammasome

**Figure 4:**
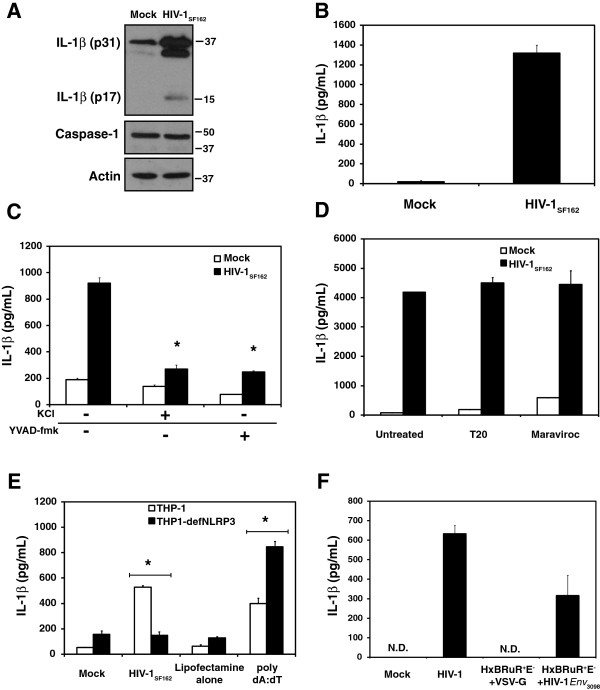
**IL-1β release in response to HIV-1**_**SF162 **_**exposure is dependent on the NLRP3 inflammasome but not viral fusion or CCR5. A.** Induction, processing and **B.** release of IL-1β from PMA-differentiated THP-1 cells following 4 hr exposure to HIV-1_SF162_. **C.** IL-1β release from PMA-differentiated THP-1 cells exposed to HIV-1_SF162_ for 4 hr +/- extracellular KCl (50 mM) or YVAD-fmk (80 μM). **D.** Release of IL-1β from PMA-differentiated THP-1 cells following 18 hr exposure to HIV-1_SF162_ +/- the inhibitor of viral fusion T20 (20 μg/mL) or the CCR5 antagonist maraviroc (100nM). **E.** IL-1β release from PMA-differentiated THP-1 cells or THP1-defNLRP3 cells exposed to HIV-1_SF162_ or poly dA:dT for 4 hr (poly dA:dT was transfected using Lipofectamine 2000). **F.** IL-1β release from PMA-differentiated THP-1 cells exposed to HIV-1_YU2,_ HxBRuR^+^E^-^ + VSVG, or HxBRuR^+^E^-^ + HIV-1_*Env*3098_ for 24 hours. Mean levels are reported for each ELISA. Bars indicate standard error. (*p < 0.05). “N.D.” indicates that IL-1β was “Not Detected”.

The above studies suggested that exposure of microglia to HIV-1 rapidly led to inflammasome-dependent cleavage and release of IL-1β. To pursue this observation further, the effects of HIV-1 on the human monocytic cell line, THP-1, were examined. Although PMA-differentiated THP-1 cells are not productively infected by HIV-1 due to high expression of SAMDH1, these cells remain permissive to viral infection [46]. Exposure of differentiated THP-1 cells to HIV-1_SF162_ but not the X4-dependent virus, HIV-1_NL4–3_, (Additional file 1: Figure S1D) for 4 hr induced expression of IL-1β, which was also processed to its mature form (Figure 4A) and released (Figure 4B) from cells. Furthermore, this response was inhibited by the addition of high extracellular K^+^ (p < 0.01), which prevents the K^+^ efflux required for activation of NLRP3 or by addition of the caspase inhibitor YVAD-fmk (p < 0.01) (Figure 4C). Importantly, the response could not be inhibited by the presence of the fusion inhibitor, enfuviride (T20), or the CCR5 antagonist, maraviroc (Figure 4D). Because the inhibitory effect of high extracellular K^+^ suggested that the NLRP3 inflammasome may be involved in the response to HIV-1, the requirement for NLRP3 in inflammasome induction by HIV-1 was subsequently tested by comparing the response of THP-1 cells with a THP-1 derived cell line that was deficient in NLRP3, THP1-defNLRP3. NLRP3 deficiency in THP1-defNLRP3 cells was confirmed by RT-PCR (Additional file 2: Figure S2). Although both THP-1 and THP1-defNLRP3 cells exhibited robust responses to the AIM2 inflammasome ligand, poly dA:dT, the response to HIV-1 was significantly reduced in the THP1-defNLRP3 cells compared with wild type THP-1 cells, supporting the notion that the NLRP3 inflammasome was integral in HIV-mediated activation and release of IL-1β (Figure 4E). Because primary microglia also responded to recombinant viral envelope protein gp120, we sought to compare the responses of THP-1 cells to pseudotyped viral particles produced through the co-transfection of HxBRuR^+^E^-^ (which lacks functional envelope gene) with either VSV-G or the HIV-1 envelope (HIV-1_Env3098_). Exposure of differentiated THP-1 cells to HxBRuR^+^E^-^ pseudotyped particles carrying HIV-1_Env3098_ induced a strong IL-1β release while cells exposed to HxBRuR^+^E^-^ carrying the VSV-G exhibited no detectable response (Figure 4F), thus confirming the importance of the HIV-1 envelope in IL-1β induction.

### HIV-1 infection induces ASC translocation in microglia

**Figure 5:**
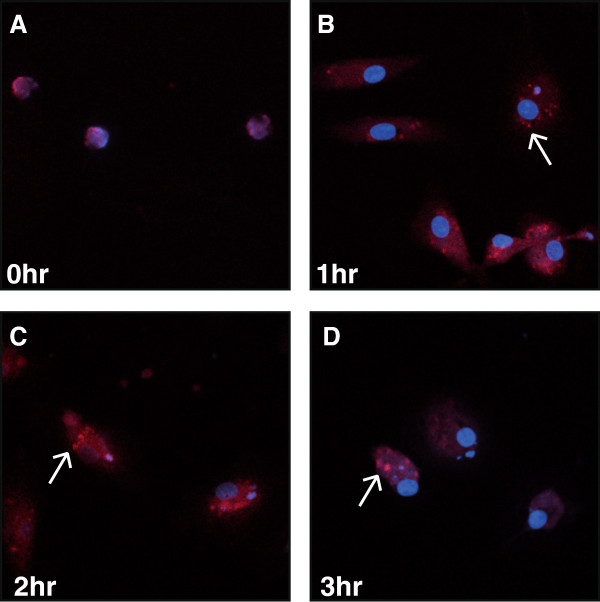
**HIV-1**_**SF162 **_**infection of human microglia induces rapid ASC intracellular translocation. A.** Mock-infected microglia exhibit minimal ASC immunoreactivity. **B.** At 1 hr post-infection ASC immunoreactivity (red) was detected in the cytoplasm. **C.** At 2 hr post-infection, punctate ASC immunoreactivity was evident in the cytoplasm of microglia. **D.** At 3 hr post-infection, aggregates of ASC immunoreactivity was readily apparent in microglia. (Original magnification 400X).

ASC represents an important protein in the formation of inflammasomes, translocating to the cytoplasm to facilitate inflammasome aggregation. To determine if ASC translocation was involved in inflammasome formation during HIV-1 infection, we investigated its expression before and during HIV-1_SF162_ infection of human microglia. These studies revealed that ASC immunoreactivity was not detected in the cytoplasm of mock-infected microglia (Figure 5A). In contrast, ASC immunoreactivity was discernible at 1 hr post-infection (Figure 5B) and increased at 2 hr post-infection (Figure 5C). By 3 hr post-infection, ASC immunoreactivity was readily apparent as a punctate appearance (Figure 5D). These findings underscored the activation of ASC during HIV-1 infection and provided further evidence of inflammasome activation in HIV-infected microglia.

### HIV-1 infection activates caspase-1 in microglia

**Figure 6:**
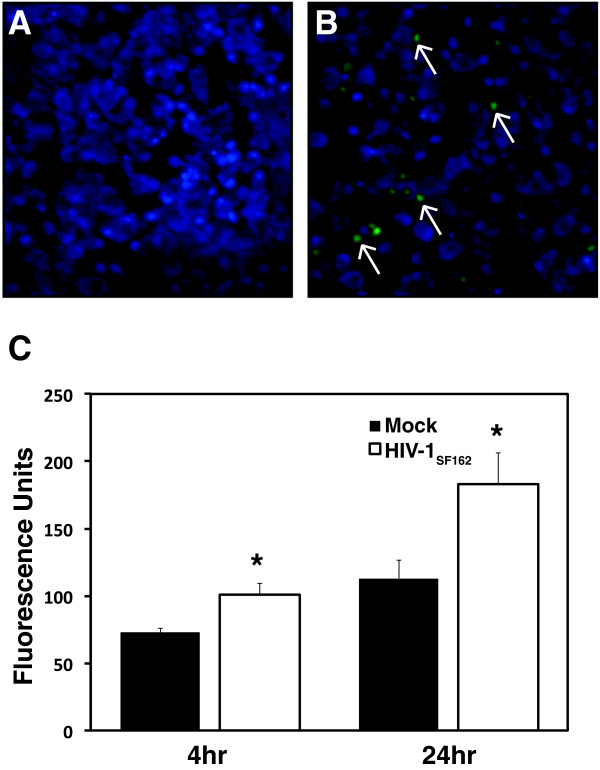
**HIV-1**_**SF162 **_**infection of human microglia activates caspase-1. A.** Caspase-1 activity was not detected in mock-infected microglial cultures that were labeled with Hoescht nuclear stain. **B.** In contrast, microglia infected by HIV-1 showed caspase-1 activity at 24 hr post-infection (Original magnification 400X). **C.** Quantitation of caspase-1 activity in microglia at 4 and 24 hr post-infection showed an increased in caspase-1 activity induced by HIV-1 infection and also over time. (Mean values reported. Bars indicate standard error. Student *t*-test, *p < 0.05).

Caspase-1 activation is a signature aspect of inflammasome assembly and action. We examined caspase-1 activity in mock- and HIV-1_SF162_-infected microglia at 24 hr post-infection, which revealed minimal caspase-1 activity (green) in mock-infected cells (Hoescht counterstained nuclei) (Figure 6A) but HIV-infected microglia exhibited abundant caspase-1 activity (Figure 6B). Quantitative analyses of caspase-1 activity in mock- and HIV-infected microglia at 4 and 24 hr post-infection showed significant increases in caspase-1 activity in HIV-infected microglia at both time points (Figure 6C). These results further verified inflammasome activation in human microglia following HIV-1 infection.

### IL-1β induction in activated microglia is associated with neurobehavioral deficits in FIV infection

**Figure 7:**
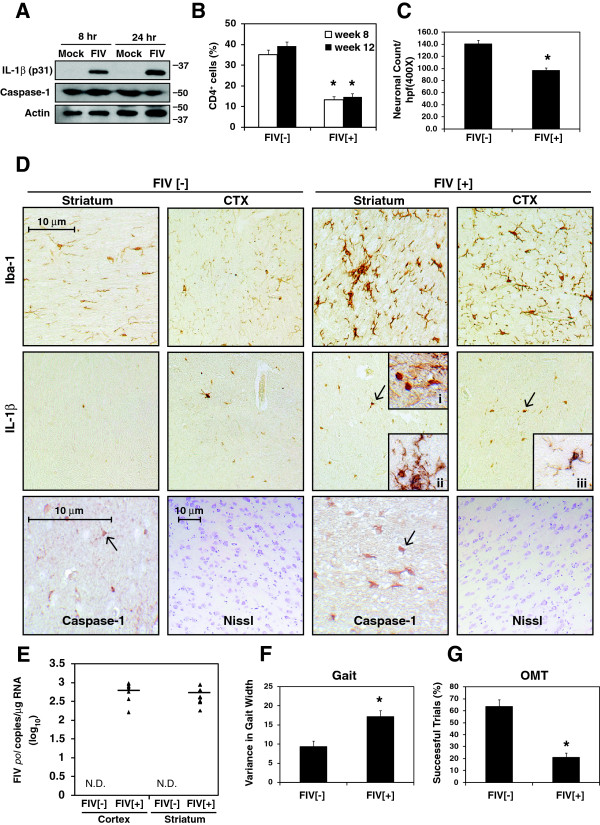
**IL-1β and caspase-1 expression in the brains of FIV-infected animals with neurologic defects. A.** IL-1β immunoreactivity was evident in feline monocyte-derived macrophages at 8 and 24 hr following FIV infection while caspase-1 expression was unaffected. **B.** CD4+ T cell count in blood of FIV-infected (n = 10) and uninfected animals (n = 6) at weeks 8 and 12 post-infection. **C.** Neuronal counts per high power field (400X) from FIV [+] animals versus FIV [-] controls at week 12 **D.** Immunohistochemical detection of Iba-1, IL-1β and caspase-1 in both striatum and cortex (CTX) from FIV [-] and FIV [+] animals (original magnification: Iba-1 and IL-1β X200; caspase-1 X600; Nissl stain, X100). Inset 'i’ shows an IL-1β immunoreactive microglial nodule. Insets 'ii’ and 'iii’ display IL-1β and Iba-1 co-immunolabeling. **E.** Detection of FIV *pol* RNA in the cortex and striatum of FIV [-] (n = 6) and FIV [+] animals (n = 10) at week 12. Performance of FIV [-] (n = 6) and FIV [+] (n = 10) animals in neurobehavioral tests of gait **(F)** and memory **(G)** at week 12 post-infection. “N.D.” indicates that virus was “Not Detected”.

The above clinical and *in vitro* observations pointed to a role for inflammasome-dependent IL-1β expression and release from microglia during HIV-1 infection. To examine the relevance of these observations in an established *in vivo* model of AIDS-associated neurologic disease, microglial activation, IL-1β expression, and expression of inflammasome components were examined in cats infected with a neurovirulent FIV strain, FIV-Ch. To confirm the direct effect of viral exposure on IL-1β expression, feline monocyte-derived macrophages were infected with FIV-Ch, showing that FIV induced IL-1β expression within 8 hr, as was observed for microglia and THP-1 cells exposed to HIV-1 (Figure 7A). *In vivo* expression was examined, revealing that in mock-infected (FIV [-]) animals, Iba-1 immunopositive microglia were observed in both striatum and cortex but the number and size (hypertrophied) of Iba-1 immunopositive microglia was increased in the FIV-infected (FIV [+]) animals (Figure 7D, row 1). IL-1β immunoreactivity was minimal in the FIV [-] striatum and cortex but was readily detected in both regions in the FIV [+] animals (Figure 7D, row 2). In fact, IL-1β immunopositive microglial nodules were evident in the FIV [+] brains (Figure 7D, inset i), which were co-localized with Iba-1 immunoreactivity (Figure 7D, inset ii). Caspase-1 immunolabeling was also present in the FIV [-] brains within cells resembling microglia (Figure 7D, row 3) but was increased on hypertrophied cells in brains from FIV [+] animals. Nissl staining of FIV [-] cortex showed numerous neurons (Figure 7D, row 3) although neuronal density was diminished in the FIV [+] brains. Neuronal counting showed that cortical neuronal density was reduced in the FIV [+] group (Figure 7C). Mean blood CD4+ T cell levels were significantly suppressed in FIV [+] animals at weeks 8 and 12 post-infection compared to FIV [-] animals (p < 0.001) (Figure 7B) and mean blood CD8+ T cell levels were elevated in FIV [+] animals at week 8 post-infection (p < 0.01) (Additional file 3: Figure S3A). In addition, viral *pol* RNA copy numbers were similar in the striatum and cortex of the FIV [+] animals but were not detected in the FIV [-] animals (Figure 7E). Neurobehavioral studies in FIV [-] and FIV [+] animals revealed that mean Gait Variance, an indicator of gait ataxia, was increased in the FIV [+] animals (p < 0.01) (Figure 7F), together with a reduction in mean performance in the Object Memory Test (OMT) (p < 0.001) (Figure 7G) compared to the FIV [-] group. Similarly, the FIV [+] group performed slower (p < 0.05) and with more errors (p < 0.01) on the Maze Tasks (Additional file 3: Figure S3B and S3C). These data indicated that microgliosis accompanied by induction of IL-1β in microglia was associated with immunosuppression and neurobehavioral deficits in this HIV/AIDS model.

### Molecular indicators of neurovirulence in FIV-infected animals

**Figure 8:**
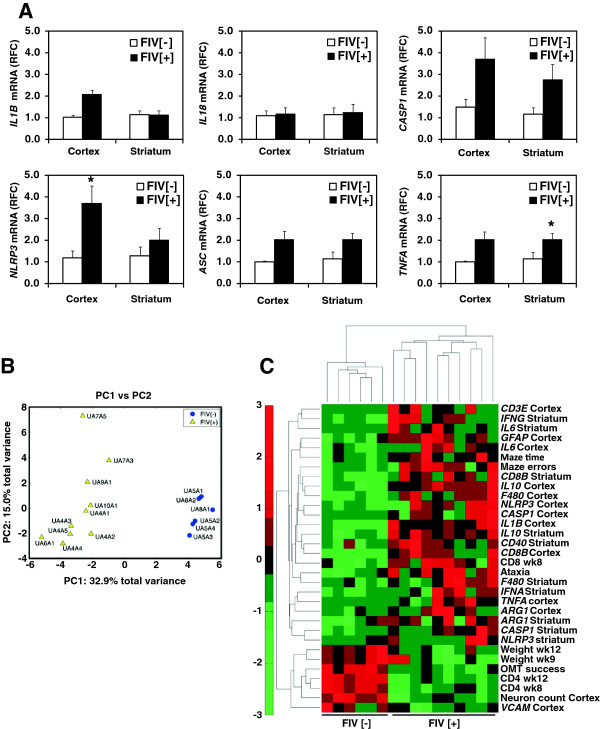
***In vivo *association of inflammasome-related genes with neurological disease in FIV [+] animals. A.** Relative fold change (RFC) in mRNA expression of inflammasome- related and immune genes in the cortex and striatum of FIV [+] (n = 6) and FIV [-] (n = 10) animals at 12 weeks post-infection. Mean RFC values are reported. Bars indicate standard error. *indicate p-value of <0.05. **B.** Principal component analysis (PCA) comparing 49 clinical, neurobehavioral and molecular variables in FIV [-] and FIV [+] animals. PC1 represents the separation of the FIV [-] and FIV [+] animals while PC2 represents intra-group variance. **C.** Hierarchical Cluster Analysis of multivariate similarities between variables and animals. Only variables that significantly contributed to PC1 (infection status) are presented in the heat map.

To examine the *in vivo* relationship between inflammasome expression and FIV infection, several inflammasome–associated genes were examined in brains from the FIV [-] and FIV [+] groups (Figure 8A). Mean *IL1B,* but not *IL18,* transcript levels were increased in the cortex of the FIV [+] group compared to the FIV [-] group (p < 0.01). *CASP1* transcript levels exhibited a trend toward increased expression in brains from FIV [+] animals while *NLRP3* transcript levels were increased in the cortex of FIV [+] animals (p < 0.05). Brain *ASC* transcript levels were similar among the FIV [-] and FIV [+] groups while *TNFA* transcript levels were increased in the striatum of FIV [+] animals (p < 0.05).

To determine the multivariate sources of variance within this *in vivo* model, principal component analyses (PCA) were performed, using 49 clinical, neurobehavioral, and molecular (cerebral cortex and striatum) variables measured in FIV [-] and FIV [+] animals, including the expression of other immune genes in the brain (Additional file 4: Figure S4 and Additional file 5: Figure S5). The first principal component (PC1) contributed 32.9% to the total variance, and therein clearly separated the FIV [-] from the FIV [+] animals (Figure 8B). The second principal component (PC2) contributed an additional 15% to total variance that was orthogonal to PC1, and identified intra-group variance. Additionally, univariate Spearman rank correlation analyses was performed (Additional file 5: Figure S5A). To investigate multivariate similarities between variables and animals, agglomerative Hierarchical Cluster Analysis (HCA) was performed. Only variables that contributed to the first PC of the PCA analysis were included in the HCA analysis as this component uniquely contributed to the separation of the two clinical groups. Thirty-two variables were identified as significantly contributing to PC1 using bootstrap re-sampling (Additional file 5: Figure S5B). The HCA is presented as a heat map with associated dendrograms (Figure 8C), revealing that multiple variables were implicated in distinguishing the FIV [-] from FIV [+] groups. The HCA heatmap showed FIV [-] and FIV [+] animals grouped into two distinct clusters, mirroring the results of the PCA analysis. Similarly, the observed variables also grouped into two distinct clusters: those positively correlated with FIV [+] and those positively correlated with FIV [-]. Within the set of variables positively correlated with the FIV [+] animals there were several sub-clusters reflecting minor heterogeneity in disease outcome. Within the context of this model, the expression of *CASP1* and *NLRP3* were highly correlated with each other both in the cortex and striatum. Cortical expression of these genes showed strong correlation to another sub-cluster of variables that included cortical *IL10*, striatal *CD8B*, *F4/80*, and maze errors. The next highest degree of correlation to these six variables was cortical *IL1B* expression. The cluster of variables that were positively correlated with FIV [-] animals included blood CD4+ T cell levels at weeks 8 and 12 post-infection, cortical neuronal counts, body weights, and performance in the object memory test. These data highlighted the complexity of the factors contributing to FIV neuropathogenesis but also implicated increased *CASP1*, *NLRP3* and *IL1B* expression in the cerebral cortex as important components of neurologic disease.

## Discussion

The current studies represent the first report of NLRP3 inflammasome-activation and release of IL-1β in response to HIV-1infection of macrophage cell types in an envelope-dependent manner. In addition, these studies constitute one of the very few studies of inflammasome expression in human brains, and the first to be complemented by similar analyses of inflammasome expression and/or activation in primary human CNS cell types including microglia, astrocytes and neurons. The present clinical, *in vitro* and *in vivo* model studies all point to brain macrophage cell types as the chief cells mediating inflammasome-associated actions. Importantly, these observations indicate that inflammasome activation by HIV-1 and the closely related lentivirus, FIV, occurred immediately after virus exposure to macrophage cells, in a caspase-1 dependent manner. Moreover, productive viral replication of cells was not a requirement for inflammasome induction, as evidenced by the capacity of HIV-1 gp120 to induce IL-1β release from microglia cells and non-replicating virus exposure to induce release from THP-1 cells. Finally, increased NLRP3, caspase-1 and IL-1β expression, particularly within the cerebral cortex were integral components of FIV-mediated neurovirulence, evident as cortical neuronal loss and complex neurobehavioral deficits, underscoring the potential importance of inflammasome activation in lentivirus neuropathogenesis.

Inflammasomes are multi-protein complexes that have gained attention for their capacity to link the sensing of infection or injury with subsequent inflammatory caspase activation and ensuing cleavage and release of the inflammatory cytokines, IL-1β and IL-18. The NLRP3 inflammasome is widely regarded as a general sensor of cellular injury or insult because the list of its activating stimuli is broad. The occurrence of specific cell physiological events, such as the lowering of intracellular K^+^, point to a shared activation pathway [47]. Another commonality shared by many NLRP3 activators (perhaps because it triggers K^+^ efflux) is the ability to destabilize endosomes following uptake. Crystalline salts, bacterial toxins and viral particles have all been reported to activate the NLRP3 inflammasome following endocytosis [48-51]. Microglia have also been reported to sense several disease-associated proteins through this pathway, including amyloid-β, mutant SOD1 and prion protein (PrP) [52-54].

The increased expression of IL-1β, IL-18 and caspase-1 observed herein among brains from HIV-1 infected persons was highly indicative of inflammasome activation during HIV-1 infection, prompting further investigation of this possibility. Previous studies have implicated inflammasome activation within the CNS as part of the response to both acute viral and bacterial infections as well as neurodegenerative disease [11-14]. However, few of these studies have examined human brains and none to our knowledge have used isolated primary CNS cells from humans. Therefore, it was imperative to characterize inflammasome expression in different CNS cell types at the outset. Along with microglia that represent the CNS resident mononuclear phagocytic cell, both neurons and astrocytes have been reported to express active inflammasome complexes under certain circumstances [12,41,55]. While not disproving those observations, a direct comparison of the expression of inflammasome-related genes between different human CNS cell types clearly highlighted microglia as the specialist innate immune cell most likely to mediate inflammasome-dependent responses. In addition to microglia, astrocytes (which outnumber microglia within the CNS by a 10 fold difference) respond to and subsequently mediate innate/inflammatory signals in the brain. Responses by activated astrocytes have been reported to include the expression of IL-1β [39-41]. However, IL-1β expression in human astrocytes at the protein level was not apparent in the current studies, even in response to strong NFκB activators such as LPS. In addition, despite detection of caspase-1 transcript in primary astrocytes the expression of caspase-1 protein was extremely low or absent from these cells, in striking difference to microglia in which caspase-1 was readily detected. The above observations prompted us to focus on investigations of HIV-1 dependent activation of the inflammasome in microglia.

Microglia are permissive to HIV-1 and productive infection can be established *in vitro*. However, as observed in the current studies, the peak production and release of virus is a temporally delayed event after IL-1β release. Thus, IL-1β induction and release were early events following HIV-1 exposure and were largely attenuated once viral production occurred (Figure 3C). These observations are consistent with studies showing that HIV-infected macrophages exhibit relatively few features of immune activation during infection, such as cytokine release [56,57]. However, innate immune sensing is expected to be an immediate response to the presence of a pathogen and indeed expression of IL-1β within human microglia occurred after a few hours with maximal IL-1β release after only 24 hr. This finding was similar to the innate immune response of plasmacytoid dendritic cells (pDC) to HIV-1 in which sensing of viral RNA by TLR7 within endosomes mediated a type 1 interferon response after overnight exposure to virus [19]. The relationship between HIV-1 and the innate immune response is assumed to contribute to subsequent pathogenesis. This assumption is exemplified by the differences observed in the transient type 1 interferon response of natural (African) non-human primate hosts to SIV infection, as compared to the chronic expression of interferons observed in Asian macaques in which SIV infection is highly pathogenic [58]. However, the pathogenic effects of a persistent response from some cells may be exacerbated by the lack of a response to HIV-1 by other innate immune cell types coupled with the ability of the same cells to transmit surface bound virus to CD4^+^ T cells in a highly efficient manner [59]. Regarding the release of IL-1β from microglia in response to HIV-1 or FIV infections and its relationship to pathogenesis, the multivariate analyses of FIV-infected animals suggested that elevated expression of caspase-1, NLRP3 and IL-1β contributed to the development of brain disease.

In West Nile Virus infection of mice, IL-1β production was reported to be protective although the response was mediated by murine neurons, which are infected by this virus [12]; HIV-1 does not infect neurons. Conversely, murine models of bacterial meningitis as well as a number of chronic neurodegenerative diseases including amyotrophic lateral sclerosis (ALS) and Alzheimer’s disease (AD) have linked inflammasome-dependent IL-1β release to the promotion of disease [11,13,53]. Gene expression profiling has identified overlap between HIV-1-associated neurocognitive disorders and other chronic neurodegenerative diseases such as Alzheimer’s and multiple sclerosis [60]. Typical features of HIV-1 infection of the brain are the occurrence of multinucleated giant cells and microglial nodules; immunohistochemical examination of the brains of SIV-infected macaques identified an enrichment of IL-1β immunopositive cells in these lesions [23]. We observed a similar enrichment in lesions in the brains of both humans and cats. In addition, it was noted in macaques that the IL-1β immunopositive cells interacting with infected cells within the microglia nodule were not themselves SIV-immunopositive [23]. These above observations lead us to the speculation that infected microglial nodules, which are repeatedly surveyed by naïve microglia with subsequent inflammasome activation and IL-1β release, could be acting as the seed site of a chronic inflammatory state that promotes cumulative CNS injury.

The specific inflammasome complex implicated in all of the aforementioned CNS diseases was the NLRP3 inflammasome. Here we report that IL-1β release in response to HIV-1 exposure is also dependent on NLRP3. This conclusion is supported by the ability of high extracellular K^+^ to inhibit the response, as well as by the attenuation of the response in a NLRP3 deficient THP-1 cell line (Figure 4C). An NLRP3-dependent response has recently been reported for monocyte-derived macrophages as well as THP-1 cells that were exposed to Hepatitis C virus (HCV) [51]. These events were mediated by the phagocytic uptake of HCV into endosomes and were independent of productive infection [50]. These authors also proposed TLR7 dependent sensing of viral RNA acted as a Signal 1 and induced the expression of IL-1β. Signal 2 was related to K^+^ efflux. The events constituting Signal 1 and 2 in response to HIV-1 remain to be elucidated. It should be noted that we could not detect IFNα release from HIV-1 exposed microglia (data not shown), as is reported to occur in pDCs [19-21] although we have previously reported on the induction of IFN-responsive genes in microglial cultures following HIV-1 exposure [61]. Arguing against a role for viral RNA and TLR7 in promoting IL-1β expression in HIV-1 exposed microglia is the observation that recombinant HIV-1 gp120 was sufficient to induce IL-1β expression and release from these cells. Soluble gp120 has been reported to exert toxic effects within the nervous system [62] and interestingly, treatment of microglial cells with gp120 has been reported to trigger an outward K + current [63]. Although the extent to which the response to soluble HIV-1 gp120 recapitulates the host response to intact live HIV-1 is limited, the induction of IL-1β expression and the NLRP3 inflammasome in response to an isolated protein is not without some precedent. For example, the bacterial protein Td92 was reported to activate the NLRP3 inflammasome through binding to the α5β1 integrin receptor [64].

## Conclusions

The present studies point to the rapid induction of IL-1β in conjunction with inflammasome activation within brain macrophage lineage cells in response to infection by HIV-1 (and FIV). These events were associated with neurovirulence, implying inflammasome activation might represent a potential therapeutic target. Future studies using proposed inflammasome inhibitors, including anti-IL-1β therapies such as anti-human monoclonal antibodies and IL-1 receptor antagonists, which have been used successfully to treat Cryopyrin-associated syndromes [65], might also be suitable for treating HIV-1 infection. Similarly, the development of new drugs such as Cytokine Release Inhibitory Drug (CRID)3 [66] and Milk fat globule-EGF 8 (MFGE8) [67], or repurposing existing drugs, such as glyburide [68] might hold promise as adjunct treatments for neurotropic infections such as HIV-1.

## Methods

### Ethics statement

The use of autopsied brain tissues is part of ongoing research (Pro00002291) approved by the University of Alberta Human Research Ethics Board (Biomedical). Written informed consent documents were signed for all samples collected. The protocols for obtaining post-mortem brain samples comply with all federal and institutional guidelines, with special respect for the confidentiality of the donor’s identity. Human fetal tissues were obtained from 15 to 20 wk aborted fetuses directly from the clinic with the written informed consent of the patient (Pro00027660) approved by the University of Alberta Human Research Ethics Board (Biomedical). All animal experiments were performed according to the Canadian Council on Animal Care (http://www.ccac.ca/en) and local animal care and use committee guidelines. The experiments involving FIV-infected cats were part of ongoing studies (AUP00000315) approved by the University of Alberta Animal Care and Use Committee.

### Reagents

Antibodies against human IL-1β, caspase-1 and actin were from Santa Cruz Biotechnology (Cat #sc-7884, sc-515, sc-1616). Anti-IL-18 was from MBL (Cat #D043-3). Anti-MHCII was from Dako (Cat #M0775). Anti-ASC was from AdipoGen (Cat #AL177). Anti-NLRP3 was from LSBio (Cat #LS-B4321). Anti-Iba-1 was from Wako (Cat #019-19741). Anti-canine IL-1β with cross-reactivity to feline IL-1β was from Kingfisher Biotech (Cat #PB0125D). The caspase inhibitor, YVAD-fmk, and the human IL-1β ELISA development kit were obtained from R and D Systems (Cat #FMK005, DY201). HIV-1 p24 antigen capture assay was obtained from Advanced Bioscience Laboratories (Cat #5421). Adenosine triphosphate (ATP) and Phorbol 12-myristate 13-acetate (PMA) were obtained from Sigma (Cat #A2383 and P8139). HIV-1 gp120 CM envelope protein (Cat #2968), AZT (Cat #3485), Efavirenz (Cat #4624), T20 (Cat #9845) and Maraviroc (Cat #11580) were obtained through the NIH AIDS Research and Reference Reagents Program, Division of AIDS, NIAID, NIH.

### Cells and viruses

THP-1 cells were cultured in RPMI (10% FBS). To differentiate, cells (5×10^5^ cells/well) were treated for 24 hr with PMA (50 nM). Following PMA treatment, cells were washed once with PBS and fresh media without PMA was added to the cells. Cells were allowed to rest for a further 24 hr without PMA before use in experiments. THP1-defNLRP3 cells (InvitroGen Cat #thp-dnlp) were treated in the same manner. THP-1 and THP1-defNLRP3 cells were transfected with 1 μg poly dA:dT using 2 μl of lipofectamine 2000 (Invitrogen Cat# 52887). Human fetal astrocytes, neurons or microglia were isolated based on differential culture conditions, as previously described [32-38,69]. Briefly, fetal brain tissues were dissected, meninges were removed, and a single cell suspension was prepared through enzymatic digestion for 30 min with 0.25% trypsin and 0.2 mg/ml DNase I, followed by passage through a 70-μm cell strainer. Cells were washed twice with fresh medium and plated in T-75 flasks coated with poly-L-ornithine at 6–8 × 10^7^ cells/flask. Cultures were maintained in MEM supplemented with 10% FBS, 2mM L-glutamine, 1mM sodium pyruvate, 1 × MEM nonessential amino acids, 0.1% dextrose, 100 U/ml Penicillin, 100 μg/ml streptomycin, 0.5 μg/ml amphotericin B, and 20 μg/ml gentamicin. For neuronal cultures, 25 μM cytosine arabinoside was added to clear the culture of proliferating cells (astrocytes). Astrocyte cultures were passaged once per week for 4–6 weeks until the neurons were eliminated. For microglial cells, mixed cultures were maintained for 2weeks at which point astrocytes and neurons formed an adherent cell layer with microglia loosely attached or free floating in the medium. Cultures were gently rocked for 20min to suspend the weakly adhering microglia in medium, which were then decanted, washed and plated. Purity of cultures was verified as previously reported by our group [32-38]. For infections and functional experiments, human microglia were plated onto 16 well glass chamber slides (70,000 cells/well) and allowed to rest for 3days prior to treatment.

The HIV-1 R5-dependent strain, HIV-1_SF162_, was produced by infecting activated human peripheral blood mononuclear cells (PBMCs) that were maintained in RPM (with human IL-1 supplementation) [34]. Day 7 or day 10 culture supernatants containing virus were centrifuged at low speeds followed by filtration through 0.22 μm filter to remove cellular debris. Supernatants were further filtered through a 100K centrifugation in filter to dialyse out small molecular weight species. The filtered viral stocks were re-suspended in OptiMEM media and the HIV-1 p24 concentration was quantified by ELISA and for experiments using microglia an inoculum (p24 20 ng/ml) was used to infect cells.

#### Production of pseudotyped viruses and infection

Pseudotyped HIV-1 virus stocks were generated by co-transfection of 293 T cells (8 × 10^6^) with 8 μg of envelope-defective HIV-1 proviral construct HxBruR^+^/E^-^ and 8 μg of vesicular stomatitis virus glycoprotein envelope (VSV-G) or HIV-1 envelope construct pSVIII-92TH014.12 (NIH AIDS Reagent #3098) using Lipofectamine 2000 (Invitrogen) according to manufacturer’s protocol. Forty-eight hours post-transfection, supernatants were collected, centrifuged at 500 × g for 10 min and filtered through 0.45 μm filters. The supernatants containing pseudotyped HIV-1 viral particles were quantified by HIV-1 p24 ELISA and used for infecting THP-1 cells. THP-1 cells (0.5 × 10^6^) cells were infected overnight with HIV-1 pseudotyped with either the VSV-G (HxBruR^+^/E^-^ + VSVG) or the HIV-1 envelope protein, (HxBruR^+^/E^-^ + HIV-1_*Env*3098_) using an innoculum of 60ng/ml HIV-1 p24*.* HIV-1_YU-2_ stocks, generated by transfection of 293 T cells with HIV-1_YU-2_ proviral DNA, were used as a positive control. Culture supernatants were collected 24h post-infection and assayed for IL-1β by ELISA. The HIV-1 envelope construct was obtained through the NIH AIDS Reagent Program, Division of AIDS, NIAID, NIH: pSVIII-92TH014.12.

### Animals

Adult pregnant cats (queens) were housed in the University of Alberta animal care facility and maintained according to the Canadian Committee of Animal Care guidelines. All queens were seronegative for feline retroviruses (FIV, FeLV). On Day 1 postpartum, animals were intracranially implanted (right hemisphere) with 200 μl of virus (10^4^ TCID_50_/mL; FIV-Ch or mock-infected). Animals were monitored daily over a 12 week period post-infection during which time body weight was measured, neurobehavioral tests were performed, and blood samples were collected. Animals were euthanized by pentobarbital overdose at 12 weeks. Tissue samples were collected and either snap frozen or fixed in 4% buffered paraformaldehyde to preserve them for subsequent analysis.

### RT-PCR

Total RNA from cells or tissue samples was isolated using TRIzol reagent (Invitrogen Cat # 15596018) and the RNeasy purification kit (Qiagen). cDNA was synthesised using superscript II reverse transcriptase (Invitrogen Cat # 100007925). Conventional PCR was visualized by agarose gel stained with Ethidium Bromide. Semi-quantitative real-time PCR was performed using SYBR green (BioRad IQ SYBR supermix) detection and the Delta-Delta CT method. Threshold cycle values for the gene of interest were normalized to GAPDH and are represented as the average relative fold change (RFC) compared with control samples.

### Immunohistochemistry

HIV/FIV [-] and HIV/FIV [+] brain tissue samples were paraformaldehyde-fixed and paraffin-embedded before sectioning and mounting. Slides were rehydrated and subjected to antigen retrieval (boiling in 10 mM sodium citrate pH6) before immunostaining. Immunoreactivity was detected using 3,3’-diaminobenzidine tetrachloride (DAB; brown) and/or 5-bromo-4-chloroindolylphosphate (BCIP; purple).

### Immunofluorescence studies

Cultured human microglia, astrocytes and neurons were seeded on an 8 well-chamber μ-slide (ibidi, Germany) and fixed with 4% paraformaldehyde in PBS for 15minutes and blocked with blocking buffer (Li-cor Biosciences) for 1 h at room temperature. Cells were then incubated with primary antibodies specific to Iba-1 (1:250, Wako), ASC (1:200, AdipoGen), MAP-2 (1:250, BD Biosciences) and GFAP (1:800, Dako) overnight at 4°C. Following washes with blocking buffer, cells were incubated with AlexaFluor secondary antibodies, anti-rabbit (647 nm, red) and anti-mouse (487, green) (1:100, Life Technologies). Cell nuclei were visualized by incubating with Hoechst nuclear dye (1:100, ImmunoChemistry Technologies) for 15minutes. Images were captured using an Olympus IX-81 confocal microscope (Quorum Technologies) using the Volocity Software.

### Caspase-1 detection assay

Human microglia were grown on a microtiter 96-well plate (50,000/well) with a clear bottom and black walls (Greiner Bio-one, Germany). Cells were mock or HIV-1_SF162_ infected following which caspase-1 activity was assayed using FAM-FLICA™ caspase-1 assay kit (ImmunoChemistry Technologies Cat#98) according to manufacturer’s protocol. Briefly, cells were incubated with FAM-FLICA reagent for 1 h at 37°C and cells were washed and incubated with media for 30 minutes to diffuse unbound FLICA. Plates were then read by setting excitation at 488 nm and emission at 530 nm using a microplate reader (Synergy™ HT, Biotek Instruments, Inc.). Adherent cells plated in 8 well-chamber slide were mock or HIV-1_SF162_ infected. At 24 hr post-infection, cells were fixed with 4% paraformaldehyde in PBS for 15minutes, following incubation with FAM-FLICA and visualised as green florescence with Olympus IX-81 confocal microscope (Quorum Technologies) using Volocity Software.

### Cell stimulation\infection

For experiments involving THP-1 or THP1-defNLRP3 cell lines, PMA differentiated cells (5×10^5^ cells/well) were exposed to HIV-1_SF162_ (20 ng/mL viral p24) or transfected with poly dA:dT (4 μg/mL). Transfections were performed using Lipofectamine 2000 reagent (7 μl/mL) (Invitrogen Cat # 52887). Samples were collected after 3 hr and collected supernatants were centrifuged to remove any cellular debris. IL-1β release was measured by ELISA.

For long term infection experiments of primary human microglia, cells (7×10^4^ cells/well) were initially exposed to HIV-1_SF162_ (HIV-1 p24, 20 ng/mL) for 24 hr at which time input virus was removed and wells were washed to remove free virus (4 × 300 μl PBS) before adding new media. Cell supernatants were subsequently collected every 3 days to determine both IL-1β and viral p24 release. Samples were analyzed by ELISA. For short term exposure experiments, microglia (7×10^4^ cells/well) were initially exposed to HIV-1_SF162_ as described above, for 18 hr before input virus was removed and new media was added to the wells. Samples were subsequently collected after allowing the cells to incubate with new media for a further 6 hr. Both cell supernatants for ELISA and cell lysates for western blot analysis were collected. For time course experiments, samples were taken at each time point following addition of the virus without further incubation.

For inhibitor treatments (i.e. YVAD-fmk, KCl, maraviroc or efavirenz), cells were pre-incubated for 1 hr at 37°C with each reagent prior to addition of infectious virus. For the viral inhibitor T20, virus was pre-incubated with T20 for 1 hr at 37°C prior to addition of virus to cells. In parallel, untreated virus was also pre-incubated at 37°C.

### Behavioral analysis of FIV infected cats

Neurobehavioral parameters in 12 week mock- (n = 6) or FIV-infected (n = 10), animals were evaluated. The behavioural tests employed have been described in detail previously [70]. Briefly, locomotor ability was determined by analyzing the inked footprints formed by cats walking across a suspended plank. Distance between the right and left paw placement was measured and the variance in gait width calculated. Spatial memory and cognitive ability was measured using a modified T-maze. The duration and number of errors to completion of the maze were recorded. The Object-Memory test was utilized to measure both spatial and object memory functions. Animals were required to step over a 6 cm moveable barrier with their forelimbs to reach a food reward. Their ability to remember the height and position of the barrier was monitored using reflective markers placed on the lateral aspects of the animal’s hindlimbs. Animal performance was recorded on video. The number of successful attempts were counted and recorded as a percent of total.

### Univariate statistical analyses

Statistical analyses of gene expression and *in vitro* cellular responses were performed using the Student *t*-test (2-tailed).

### Multivariate data analyses

Data for each variable was converted to z-scores to allow unbiased analysis. Missing data values were imputed using the standard *k*-nearest neighbor (*k*-NN) methodology (k = 3) [71] Principal components analysis (PCA) was performed to investigate multivariate correlation within the data [72]. PCA is a mathematical procedure, which enables the correlation between the N observed variables to be projected onto a smaller set of linearly uncorrelated latent variables called Principal Components (PCs). This PCA projection summarizes the predominant patterns in the multivariate data. The PCs are calculated such that the first PC describes the direction of maximum variance in the multivariate data and each subsequent PC in turn describes the next highest orthogonal (uncorrelated) direction variance in the data. A PCA score plot is a projection of the original data set onto the PC axes, with each point representing a single animal. Clustering of points indicates a strong correlation (i.e. similar variable profile). Variables that contribute significantly to each axis of the PCA projection can be readily determined using bootstrap re-sampling [73].

To assess the multivariate similarities of those variables significantly contributing to the PCA, unsupervised 2-way Agglomerative Hierarchical Cluster Analysis (HCA) was performed [74]. This algorithm used a multivariate Euclidean distance metric and Ward’s group linkage to generate the 2-way hierarchical cluster trees; it clustered first with respect to animals and then to variables. The results were displayed as a heatmap with associated cluster dendrograms; the lower the linkage in the dendrogram, the more similar the feature.

## Competing interests

The authors declare that they have no competing interests.

## Authors’ contributions

Conceived and designed experiments: JGW, CP. Performed the experiments: JGW, MM, FM, BAM, WGB, CP. Analyzed data: JGW, SNR, MM, BAM, FM WGB, DIB, CP. Computational and statistical analyses: SNR, MM, DIB. Wrote paper: JGW, CP. All authors read and approved the final manuscript.

## Supplementary Material

Additional file 1: Figure S1A Semi-quantitative real-time PCR showing the relative fold change in expression in THP-1 cells and primary human microglia or astrocytes. **B.** IL-1β expression in microglia following infection with HIV-1_SF162_. Prior to infection, cells were pre-treated with buffer control or with AZT (10 μg/mL) or Efavirenz (1 μg/mL). **C.** IL-1β release from microglia following infection with HIV-1_SF162_. Prior to infection, cells were pre-treated with buffer control or cytochalasin D. **E.** IL-1β release from PMA-differentiated THP-1 cells exposed to HIV-1_SF162_ or HIV-1_NL4–3_ for 4 hr. Click here for file

Additional file 2: Figure S2A NLRP3 mRNA expression in THP1-defNLRP3 cells relative to conventional THP-1 cells with and without PMA differentiation of cells. Click here for file

Additional file 3: Figure S3A CD8+ T cell levels in blood of FIV [-] and FIV [+] animals at week 8 and week 12 post-infection. **B.** and **C.** Performance of FIV [-] and FIV [+] animals in neurobehavioral tests at week 12 post-infection. **D.** Mean weights of FIV [+] and FIV [-] animals over 12 weeks. Click here for file

Additional file 4: Figure S4 Relative fold change in mRNA expression of inflammation-related genes in the cortex or striatum of FIV [+] cats versus FIV [-] controls. Click here for file

Additional file 5: Figure S5A Univariate Spearman rank correlation analysis of 49 clinical, neurobehavioral and molecular variables in FIV [-] and FIV [+] animals. **B.** Bootstrap re-sampling to determine which factors in the Hierarchical Cluster Analysis significantly contribute to PC1 (infection status). Click here for file
