# Supplementary figures and images for: Rapid inflammasome activation in microglia contributes to brain disease in HIV/AIDS
Source: Retrovirology. 2014 May 13;11:35. doi: 10.1186/1742-4690-11-35 (PMC4038111; doi:10.1186/1742-4690-11-35)

**Figure S1**

**A**

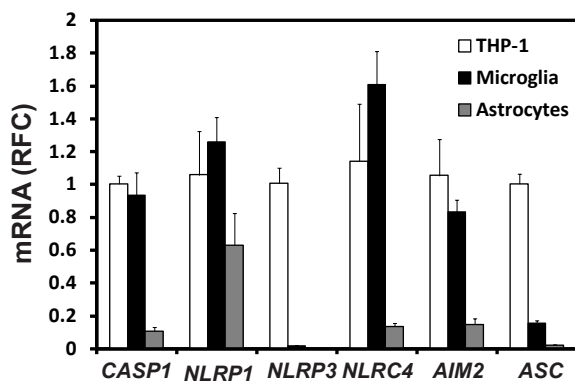

**B**

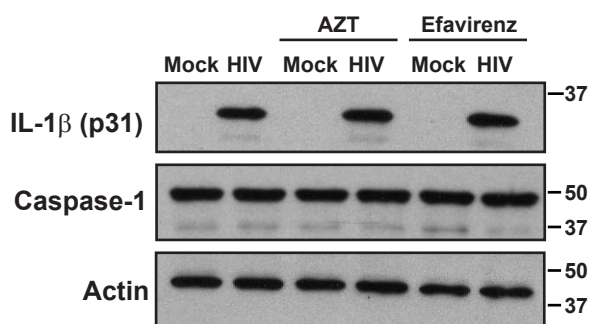

**C**

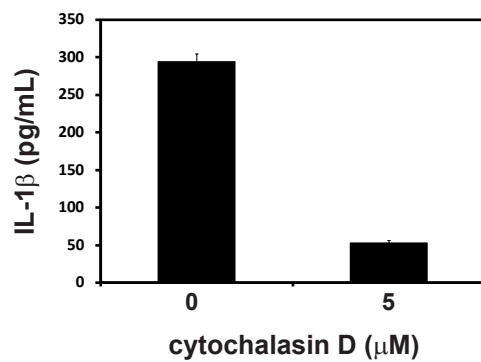

**D**

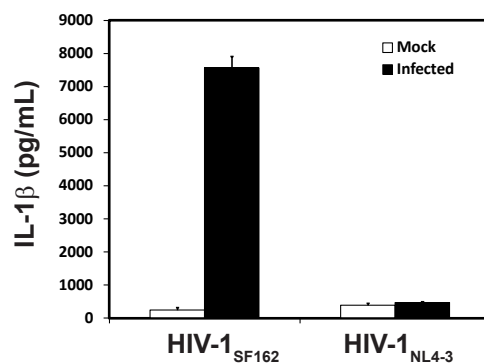

Supplement: Additional file 1: Figure S1A — Semi-quantitative real-time PCR showing the relative fold change in expression in THP-1 cells and primary human microglia or astrocytes. B. IL-1β expression in microglia following infection with HIV-1SF162. Prior to infection, cells were pre-treated with buffer control or with AZT (10 μg/mL) or Efavirenz (1 μg/mL). C. IL-1β release from microglia following infection with HIV-1SF162. Prior to infection, cells were pre-treated with buffer control or cytochalasin D. E. IL-1β release from PMA-differentiated THP-1 cells exposed to HIV-1SF162 or HIV-1NL4–3 for 4 hr. [file 1742-4690-11-35-S1.pdf]

Figure S2

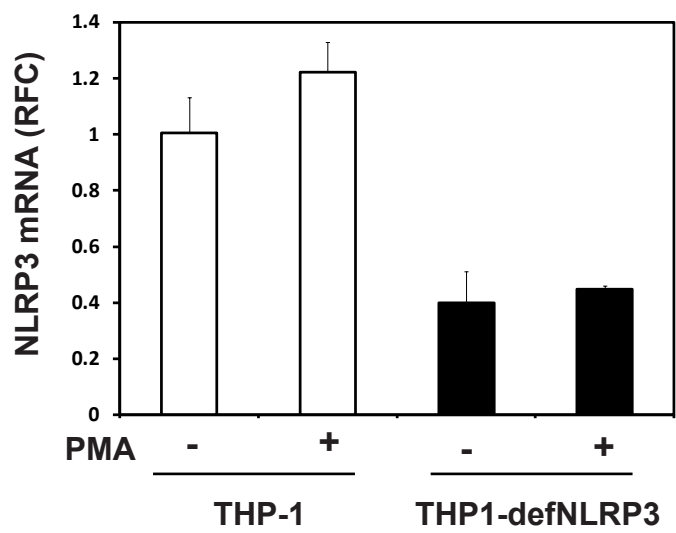

Supplement: Additional file 2: Figure S2A — NLRP3 mRNA expression in THP1-defNLRP3 cells relative to conventional THP-1 cells with and without PMA differentiation of cells. [file 1742-4690-11-35-S2.pdf]

Figure S3

A

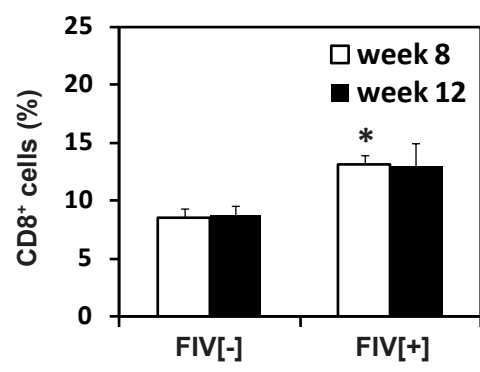

B

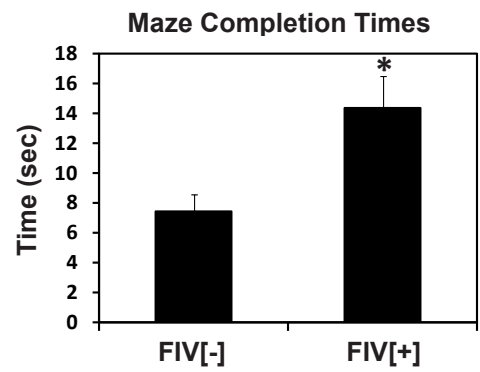

C

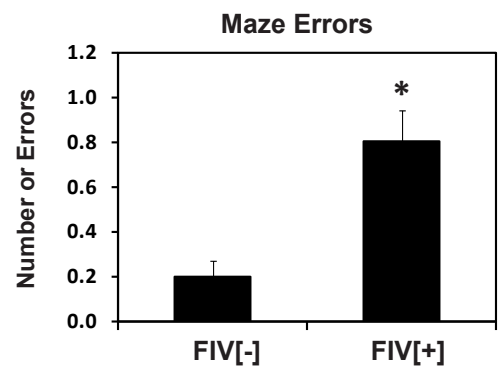

D

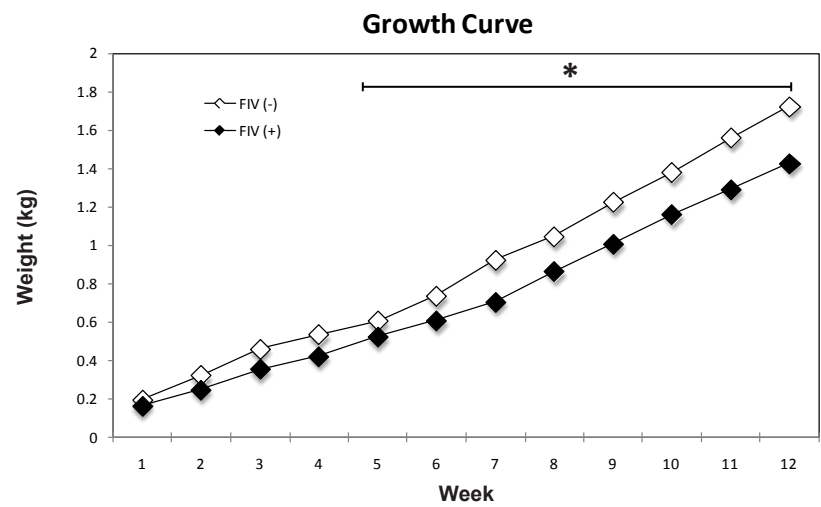

Supplement: Additional file 3: Figure S3A — CD8+ T cell levels in blood of FIV [-] and FIV [+] animals at week 8 and week 12 post-infection. B. and C. Performance of FIV [-] and FIV [+] animals in neurobehavioral tests at week 12 post-infection. D. Mean weights of FIV [+] and FIV [-] animals over 12 weeks. [file 1742-4690-11-35-S3.pdf]

Figure S4

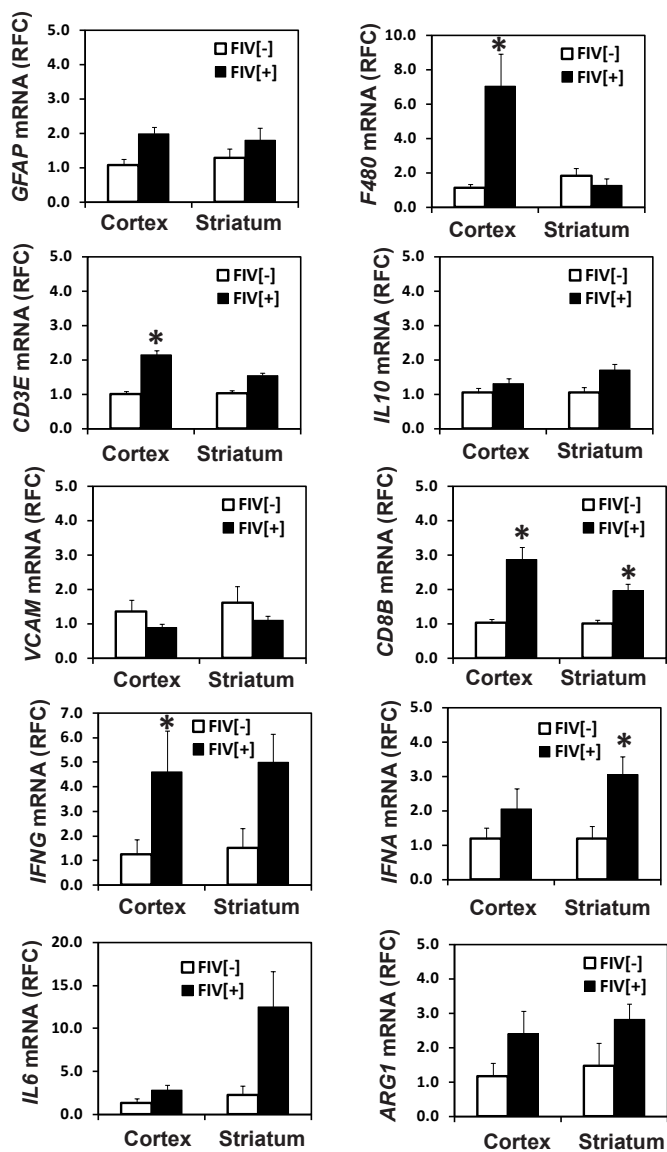

Supplement: Additional file 4: Figure S4 — Relative fold change in mRNA expression of inflammation-related genes in the cortex or striatum of FIV [+] cats versus FIV [-] controls. [file 1742-4690-11-35-S4.pdf]

Figure S5

A

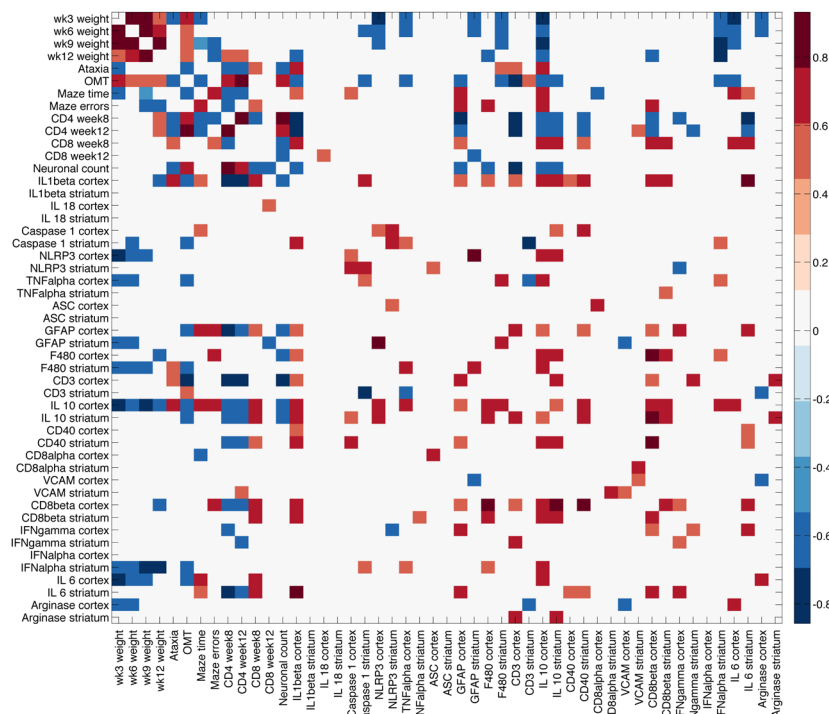

B

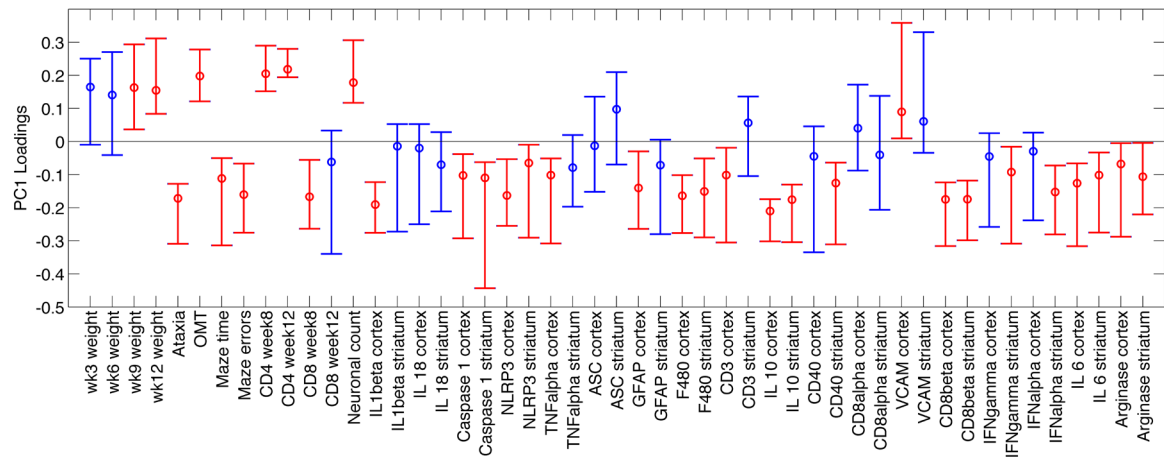

Supplement: Additional file 5: Figure S5A — Univariate Spearman rank correlation analysis of 49 clinical, neurobehavioral and molecular variables in FIV [-] and FIV [+] animals. B. Bootstrap re-sampling to determine which factors in the Hierarchical Cluster Analysis significantly contribute to PC1 (infection status). [file 1742-4690-11-35-S5.pdf]
